# Emerging Therapies for Antibody-Mediated Rejection in Kidney Transplantation

**DOI:** 10.3390/jcm12154916

**Published:** 2023-07-26

**Authors:** Farah Abuazzam, Casey Dubrawka, Tarek Abdulhadi, Gwendolyn Amurao, Louai Alrata, Dema Yaseen Alsabbagh, Omar Alomar, Tarek Alhamad

**Affiliations:** 1 Division of Nephrology, Department of Medicine, Washington University School of Medicine, St. Louis, MO 63110, USA; faraha@wustl.edu (F.A.); atarek@wustl.edu (T.A.); amurao@wustl.edu (G.A.); louai@wustl.edu (L.A.); dema@wustl.edu (D.Y.A.); aomar@wustl.edu (O.A.); 2 Department of Pharmacy, Barnes Jewish Hospital, St. Louis, MO 63110, USA; casey.dubrawka@bjc.org; 3 Transplant Epidemiology Research Collaboration (TERC), Institute of Public Health, Washington University School of Medicine, St. Louis, MO 63110, USA

**Keywords:** kidney transplant, allograft rejection, T-cell mediated rejection, antibody-mediated rejection, mix rejection, transplant immunology, kidney graft function

## Abstract

Despite the advances in immunosuppressive medications, antibody-mediated rejection (AMR) continues to be a major cause of kidney allograft failure and remains a barrier to improving long-term allograft survival. Recently, there have been significant advances in the understanding of the pathophysiological process of AMR, along with the development of new therapeutic options. Additionally, surveillance protocols with donor-derived cell-free DNA and gene profile testing have been established, leading to the early detection of AMR. A multitude of clinical trials are ongoing, opening numerous opportunities for improving outcome in kidney transplant recipients. In this brief review, we discuss the emerging therapies for managing both active and chronic active AMR and highlight the ongoing clinical trials.

## 1. Introduction

Antibody-mediated rejection (AMR) was first formally described as a clinicopathological diagnosis in the Banff 97classification [1] and is recognized as a major cause of acute and chronic kidney allograft dysfunction with an eventual progression to allograft failure [2]. Over the last twenty years, several studies have shed light on the mechanisms of injury in AMR, leading to an evolved understanding of the pathophysiological processes contributing to the complex spectrum. This, along with the establishment of surveillance protocols, has led to advances in AMR detection and the development of new potential therapies. However, this progress has not been matched in long-term allograft survival. The incidence of graft failure remains high at 4% per year after the first year of transplantation [3,4], and the rate of the improvement has plateaued since the late 1980s [5].

The treatment of AMR has evolved over the past two decades. Due to the many contributing pathways of injury, a multimodal treatment approach is necessary. Current 2009 KDIGO Guidelines [6], along with published consensus guidance, recommend treatment of AMR in kidney transplant recipients using one or more of the following, with or without the addition of corticosteroids, therapeutic plasmapheresis (TPE), intravenous immunoglobulin (IVIG), an anti-CD20 antibody, most commonly rituximab, or a lymphocyte-depleting antibody therapy, along with the optimization of the maintenance immunosuppression regimen [7]. However, these conventional therapeutic modalities focusing on the removal and neutralization of the alloantibodies in addition to the attenuation of the immune response to alloantibodies have failed to significantly alter the long-term poor outcomes and are further limited by variable success rates. The management of AMR continues to pose a significant challenge to allograft survival in kidney transplant recipients. This is further highlighted by the absence of any FDA-approved medications for the treatment of AMR, necessitating the advent of novel therapies. In this article, we review the emerging therapies for managing AMR in kidney transplantation along with the ongoing clinical trials.

## 2. Methods

In this review, we searched PubMed and clinicaltrials.gov to collect information about emerging therapies for AMR, both chronic and acute. We added medications that are either in a current clinical trial (regardless of the phase) or with a promising mechanism that would likely end up in a clinical trial in the near future.

## 3. Carfilzomib

**Figure 1:**
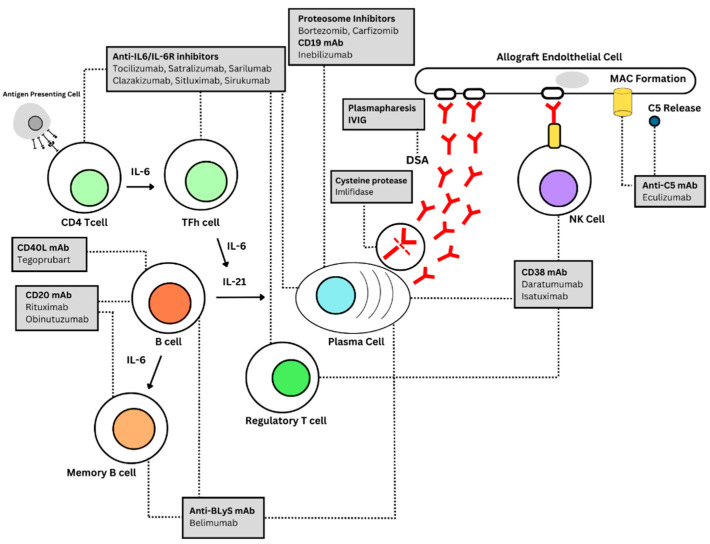
This figure shows the pathogenesis of AMR wherein an alloantibody reaction is presented by the antigen-presenting cell (APC) to the CD4 T-cell, which later matures to TFh helper cells amplifying B-cells through IL-6/21 activity. This leads to B-cell proliferation and differentiation, which in turn generates antibody-producing plasma cells. The binding of alloantibodies to the allograft endothelial cell induces natural killer (NK) cells (and macrophage) activation and triggers complement activation through the classical pathway (CP) [12]. IL-6 and IL-6R monoclonal antibodies (mABS) such as clazakizumab may prevent proper B-cell activation/differentiation and affect the production of plasma cells. IL-6 antagonists may also enhance regulatory T-cell formation, which inhibita T-cell proliferation and cytokine production and play a critical role in preventing autoimmunity [13]. CD38 mAbs and proteosome inhibitors may decrease alloantibody-producing plasma cells and may also affect NK cells and regulatory T-cells [13]. A CD40L monoclonal antibody, such as Tegoprubart, plays an important role in the amplification of immune response and the production of antibodies and may play a role in AMR and rapid allograft loss [14]. Eculizumab is a long-acting humanized mAb targeted against complement C5 which inhibits the deployment of the terminal complement system, including the formation of MAC [15]. Both plasmapheresis and IVIG help combine the removal of circulating DSA with the immunomodulation of the antigraft immune response, specifically B-cell response, while the multimodal function of intravenous immunoglobulin includes interference with B- and T-cell activation, antibody formation and recycling, as well as complement activation [16]. Imlifidase, an IgG-degrading enzyme of streptococcus pyogenes, may rapidly reduce or even eliminate anti-HLA DSA, and is currently undergoing clinical trials in AMR. This enzyme cleaves human IgG at a highly specific amino acid sequence and effectively blocks complement-dependent cytotoxicity (CDC) and antibody-dependent cellular cytotoxicity [7]. CD20 mAbs such as rituximab are B-cell depleting and promote cell lysis by triggering the complement of both CDC and ADCC [13]. The anti-B-lymphocyte stimulator (BlyS)-specific mAb belimumab may inhibit the survival of B-cells that causes chronic inflammation and tissue damage [16].

Carfilzomib is a second-generation irreversible proteasome inhibitor. Its mechanism of action is similar to that of Bortezomib (Figure 1). It has been used off-label in relapsed or refractory MM as a single or combination therapy. Bortezomib is a proteasome inhibitor that is FDA-approved for the treatment of multiple myeloma (MM) [8] and has been used off-label as a desensitizing agent in sensitized kidney transplant recipients [9]. It exerts its effect by inducing apoptosis of bone-marrow-derived plasma cells, the main antibody-producing cells, thereby leading to the reduction in alloantibodies production in sensitized recipients [10]. A multitude of studies have analyzed the effectiveness of Bortezomib in AMR in the last decade. The adverse events reported in these studies include fatigue, diarrhea, mild cases of peripheral neuropathy, thrombocytopenia, neutropenia, and viral and bacterial infections [11]. Additionally, there were reported cases of death secondary to septic shock.

One issue with bortezomib was the rebound injury. A case series involving 23 patients with acute AMR, of whom 11 had refractory AMR, received 1 to 3 cycles of Bortezomib. The study shows an improvement in the serum creatinine (Scr) at the end of therapy (2.4 mg/dL) which was noticed compared with the Scr (2.9 mg/dL) at the beginning of the study, but this improvement was not sustained at follow-up. A lack of histological response was reported in 48% of patients. Based on this result, the authors conclude a poor response of later AMR to both conventional and Bortezomib therapies [17].

In terms of carfilzomib, six nonhuman primate kidney transplant recipients used carfilzomib and belatacept in the desensitizing protocol. It showed a significantly prolonged graft survival and reduced AMR scores on renal biopsy at 1 month. However, four of five animals of long-term graft survival showed the gradual rebound of DSA and AMR [18].

Its effectiveness in humans was studied only in lung and heart transplant recipients who had acute AMR with promising results [19,20]. But there are no data yet for its use in managing AMR in human kidney transplant recipients.

A study of its safety and toxicity showed an acceptable profile as a desensitizing agent [21]. The adverse events reported in human transplant recipients include acute kidney injury and thrombocytopenia [22]. There was a reported death secondary to chronic rejection, infection, pulmonary embolism, and malignancy [20].

Overall, carfilzomib is a promising agent. Future studies are underway, such as clinical trials in organ transplantation (CTOT-42), to evaluate the efficacy of carfilzomib for the treatment of chronic active AMR.

## 4. Interleukin-6 Targeted Therapies

### 4.1. Tocilizumab

Tocilizumab is the first in-class recombinant humanized monoclonal antibody targeted at the interleukin-6 receptor (IL-6R) [23]. It binds to both soluble and membrane-bound forms of IL-6R to inhibit signaling through these receptors. IL-6 is an inflammatory cytokine which plays a critical role in B- and T-cell activation, proliferation, and function. Additionally, IL-6 has been recognized in key signaling pathways associated with rejection in transplant recipients [24]. Tocilizumab is FDA-approved for the treatment of rheumatoid arthritis and juvenile idiopathic arthritis [23].

Patients treated with tocilizumab are at increased risk of serious infection, including tuberculosis, invasive fungal infections, and bacterial, viral, or other opportunistic infections. Additionally, gastrointestinal perforation and elevation in transaminases have been reported complications [23].

**Figure 2:**
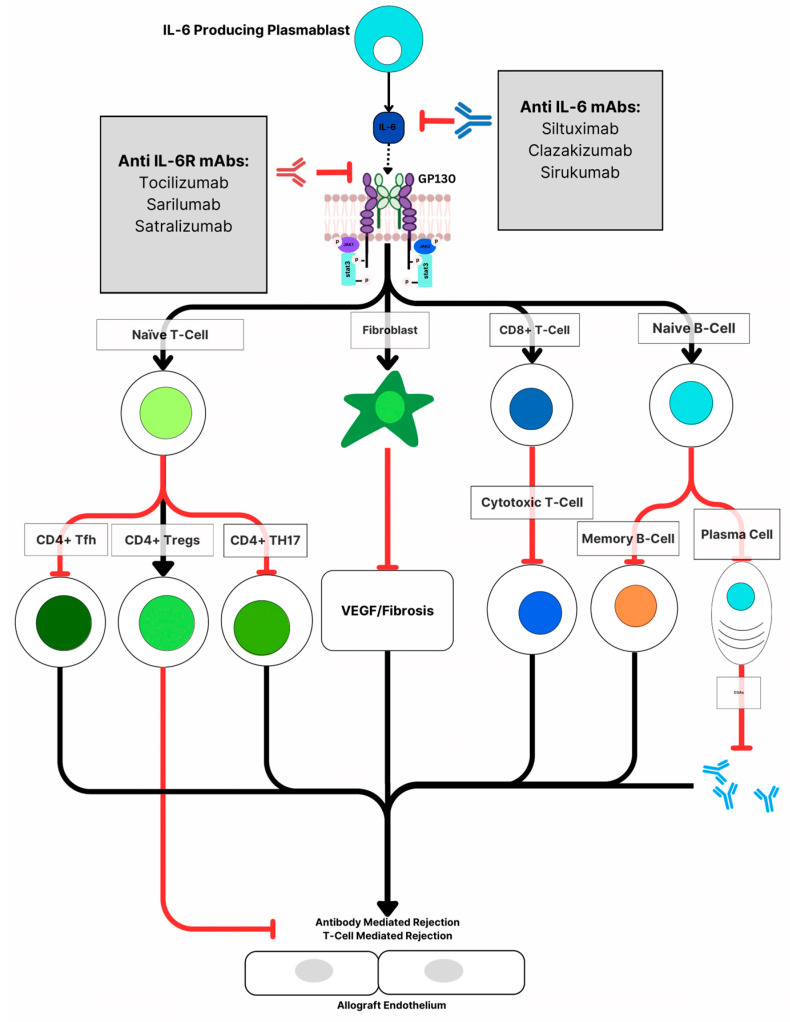
IL-6 inhibitors can block the action of IL-6 either directly or at the receptor level, the blockage of IL-6 leads to inhibition of the maturation of Naïve CD4+ T-Cells into CD4+ Tfh and TH17, which inhibits autoimmunity and decreases antibodies production, and upregulates differentiation into CD4+ Tregs, which suppresses inflammatory response. The blockage of IL-6 also inhibits the differentiation of Naïve B-Cells to DSAs producing plasma cells and memory B-Cells.

Earlier studies examined the use of tocilizumab in desensitization. A phase I/II trial reported a strategy of using tocilizumab and IVIG as desensitizing agents in sensitized kidney transplant recipients. The study reported this strategy as a safe and effective in reducing the DSA titer and prevention of AMR [25] (Figure 2).

While the available literature is limited for its use in the treatment of acute AMR, Pottebaum et al. evaluated the use of tocilizumab in addition to conventional therapy in seven kidney transplant recipients with acute AMR. Renal function improved or stabilized during the treatment, and a ≥50% reduction in donor-specific antibody (DSA) titers in four patients was noted [26]. However, recurrent rejection has been noticed in several patients, in particular, for patients who received less than 6 months therapy of tocilizumab.

A multitude of studies evaluating tocilizumab in chronic active AMR have been published with promising results. In a study of 36 kidney transplant recipients with chronic active AMR who failed conventional therapy, tocilizumab was offered as a rescue therapy for 6–25 months. Significant reductions in DSA titers and the stabilization of renal function were seen at 2 years in tocilizumab patients, along with graft and patient survival rates of 80% and 91%, respectively, at 6-year follow-up. No significant or severe adverse events were reported [27]. Additionally, Noble et al. conducted a retrospective study in 40 kidney transplant recipients who received monthly tocilizumab for chronic active AMR. At 12 months follow-up, no clinical or histological worsening was seen, except for those whose clinical condition was more severe at the time of initiation [28].

In Lavacca et al., tocilizumab adopted as first-line therapy in 15 kidney transplant recipients with chronic active AMR was associated with the stabilization of glomerular filtration rate (GFR) and proteinuria, a significant reduction in DSA titers, and histological improvement on the protocol biopsies after 6 months despite advanced transplant glomerulopathy in most patients at the time of the study’s initiation [29].

On the contrary, conflicting findings have been reported by Kumar et al. who evaluated the efficacy and safety of tocilizumab in 10 kidney transplant recipients with chronic active AMR. The patients received tocilizumab for a median of five doses. No improvements in renal function, change in the slope of eGFR decline, or histological improvement on the protocol biopsies were noted at a median follow up of 12 months. There was one patient death due to a complication from hip infection [30].

### 4.2. Clazakizumab

Clazakizumab is a genetically engineered, high-affinity, humanized monoclonal IgG1 antibody that binds IL-6. It is the most potent and longest acting in the IL-6/IL-6R blocking category [31]. Clazakizumab’s anti-inflammatory potency was monitored in sensitized kidney transplant recipients showing an effective reduction in inflammatory markers and cytokines [32] (Figure 2). To this point, clazakizumab remains strictly investigated in its use, and along with use in solid organ transplantation, has also been evaluated in rheumatoid and psoriatic arthritis.

In a phase 2 single-center open-label study, 10 kidney transplant recipients with refractory active AMR received clazakizumab monotherapy monthly for 12 months. Clazakizumab treatment showed a trend toward the stabilization of eGFR and reductions in DSA titers and graft inflammation [33]. Based on these findings, a large phase 3 placebo-controlled clinical trial (IMAGINE) of clazakizumab in chronic active AMR is currently recruiting participants across North America, Europe, Asia, and Australia (Further details in Table 1).

Doberer et al. published a pilot randomized controlled trial evaluating the safety and efficacy of clazakizumab in late AMR. Twenty patients with positive DSA after 1 year of kidney transplant were randomized to receive clazakizumab or placebo weekly for 12 weeks (part A). After a period of 12 weeks, the mean eGFR decline was slower and the DSA titers were significantly decreased in the clazakizumab group. Subsequently, all patients received clazakizumab for 40 weeks (part B). As a result, the slope of eGFR decline improved in those patients. However, 25% of patients developed serious adverse events while on treatment, including diverticulitis, pleural effusions, and acute kidney injury [34].

However, Mayer et al. recently published a secondary endpoint analysis of the effect of clazakizumab treatment on biomarkers indicative of tissue damage (donor-derived cell-free DNA) and parenchymal inflammation (C-X-X motif chemokine ligand) in 20 kidney transplant recipients with late AMR [35]. Patients were randomized to treatment with clazakizumab or matching placebo for 12 weeks (part A), followed by all patients receiving clazakizumab for 52 weeks (part B). As a result, there was no association between biomarkers levels and the resolution of molecular and morphological AMR activity; however, the subtle response might be overlooked by early biomarkers surveillance.

In summary, clazakizumab comes with a hope of less rebounding antibodies and rejection than prior IL-6 receptor antibodies.

The other IL-6 inhibitors include sitluximab and sirukumab; they are new agents that have not been studied in the context of kidney transplant [36,37].

### 4.3. Daratumumab

Daratumumab is an immunoglobulin G1K human monoclonal antibody. It is the first in-class human-specific therapy to target CD38-expressing plasma cells. CD38-expressing cells also include regulatory T- and B-cells. Daratumumab is an FDA-approved antibody for newly diagnosed or relapsed/refractory multiple myeloma [38]. Daratumumab exerts its immunomodulatory effect through several Fc-dependent immune effector mechanisms, including antibody-dependent cellular phagocytosis, antibody-dependent cell-mediated cytotoxicity, complement-dependent cytotoxicity, and direct cellular apoptosis [39] (Figure 1).

Reported adverse events with daratumumab include infusion-related reaction, volume overload, hypogammaglobulinemia, myelosuppression, gastrointestinal upset, and infection [38].

Of note, it is also reported that daratumumab may interfere with cross-matching and red blood cell (RBC) antibody screening as it binds to CD38 on RBCs and results in a positive indirect Coombs test. Another important consideration during daratumumab therapy is the potential for resistance, mainly through complement-inhibitory proteins, CD55 and CD59, and the development of antidrug antibodies [40].

Kwun et al. conducted a study to evaluate Daratumumab therapy as a desensitizing agent in sensitized nonhuman primate model for the management of AMR in two clinical combined heart–kidney transplant recipients. The result of the study showed a significant reduction in DSA titer in the nonhuman primate model and transplant clinical cases when it was used as a desensitizing agent and for the management of AMR. However, the reduction in DSA titer was not maintained in the nonhuman primate as all recipients demonstrated the rapid rebound of antibodies [41].

Multiple case reports over the past 5 years have highlighted promising results of daratumumab use in the treatment of acute AMR. Spica et al. reported a case of its use in refractory AMR in an ABO-incompatible kidney transplant recipient. After failed conventional treatment, the patient received daratumumab as rescue therapy. The blood group antibody titer decreased and remained at low levels and allowed the graft function to recover [42].

Recently, a case report of daratumumab use in two kidney transplant recipients with late or chronic active AMR was published. The patients were treated using a novel regimen of early intensive therapy with daratumumab plus TPE and IVIG and later maintenance therapy with daratumumab alone. A remarkable decrease in DSA titers was noticed. After 20 months of follow-up, both patients maintained low DSA titers and normal/improved renal function while on maintenance. However, one patient deteriorated because of acute T-cell-mediated rejection [43].

Currently there are 304 clinical trials testing daratumumab in diverse pathological conditions, 1 of which includes an evaluation of the efficacy of daratumumab in kidney transplant as a desensitizing agent [44] (Further details in Table 1).

Isatuximab is another CD38 inhibitor; it has been approved for use in multiple myeloma [45]. There have not been any trials on isatuximab for use in kidney transplant.

### 4.4. Belimumab

Belimumab is a humanized anti-B lymphocyte stimulator (BLyS) IgG1 monoclonal antibody. It is approved by the FDA for the management of systemic lupus erythematosus and has shown therapeutic efficacy in lupus nephritis [46]. BlyS, also known as B-cell activating factor (BAFF), is a cytokine of the tumor necrosis factor (TNF) family that promotes B-cell maturation, survival, and activation. The overexpression of BLyS leads to autoimmunity. In kidney transplant recipients, high serum concentration of BLyS have been associated with the development of de novo DSA and increased risk of AMR [47,48] (Figure 1).

Gastrointestinal upset, dizziness, infection, depression, and diabetes have been reported adverse events related to belimumab therapy in kidney transplant recipients. Cases of progressive multifocal leukoencephalopathy have also been reported [49,50].

Belimumab has been studied for the prevention of AMR in a phase 2 randomized controlled trial of 28 kidney transplant recipients randomized to belimumab or matching placebo in addition to standard immunosuppression therapy of basilixmab, mycophenolate, tacrolimus, and prednisone. The study concluded the effectiveness of belimumab with the standard therapy with no increased risk of infection compared to placebo [51].

The CAMPBEL open-label pilot study aimed to evaluate belimumab therapy in addition to the standard immunosuppression therapy of alemtuzumab, and steroid induction with mycophenolate and tacrolimus maintenance in kidney transplant recipient was terminated because of recruitment complications due to the COVID-19 pandemic [52].

Belimumab therapy in kidney transplant recipients with active AMR has not been well studied. In a case report of a combined kidney–pancreas transplant recipient with mixed rejection of the kidney allograft, belimumab was utilized for persistent DSA and inflammation following conventional treatment with TPE, rituximab, and IVIg. The patient was noted to have a reduction in class II DSA and improved graft function [53,54].

### 4.5. Imlifidase

Imlifidase is a novel recombinant cysteine protease derived from *Streptococcus pyogenes* which is able to cleave human IgG antibodies at the lower hinge region of the IgG heavy chain. Subsequently, this cleavage leads to the inhibition of complement-dependent cytotoxicity and antibody-dependent cellular cytotoxicity, the cleavage of B-cell receptors, and a reduction in natural killer (NK) cell activity [55] (Figure 1).

Imlifidase has primarily been evaluated and is currently being assessed in ongoing studies as part of desensitization protocols [56]. Based on promising data in sensitized patients, a multicenter randomized clinical trial is currently recruiting patients to assess the efficacy of imlifidase versus TPE for the management of chronic or active AMR in kidney transplant recipients [57].

While imlifidase remains a promising novel treatment option for the management of AMR, its use may be limited by risk for antibody rebound, including anti-imlifidase antibodies, DSA, and total IgG. Understanding this risk remains insufficient; however, it may potentially be able to be mitigated by the administration of anti-CD20 antibody and/or IVIg. Furthermore, a number of important drug–drug interactions exist. Because imlifidase cleaves IgG antibodies, co-administered antibody-based therapies may also be impacted. Consideration should be given to the appropriate separation of administration with IVIg for 12 h, rituximab, basiliximab, rabbit antithymocyte globulin, and alemtuzumab for 4 days, and belatacept for 1 week to prevent a reduction in the efficacy of concomitant therapies [55].

## 5. New CD-20 Agents

Numerous studies assessing the efficacy of anti-CD20 monoclonal antibody in the treatment of antibody-mediated diseases have been published. Of the anti-CD20 antibody therapies, rituximab remains a well-established agent utilized in solid organ transplantation and is commonly employed in the current strategy for AMR treatment in kidney transplant recipients.

### 5.1. Ofatumumab

Ofatumumab is an alternative anti-CD20 agent that is approved by the FDA for the treatment of relapsing multiple sclerosis (Figure 1). A case report of a sensitized combined heart–kidney transplant recipient who failed her first heart transplant due to cardiac allograft vasculopathy was treated with TPE, IVIG, ofatumumab, and tocilizumab as desensitization therapy. During the first-year follow-up after transplant, she maintained good cardiac and kidney grafts function. No episodes of rejection were reported, but she did develop persistent BK viremia [58].

### 5.2. Obinutuzumab

Obinutuzumab is a glyco-engineered type II anti-CD20 monoclonal antibody. When compared to rituximab, a type I anti-CD20 antibody agent and the current standard of care in AMR treatment, obinutuzumab has been shown to offer more profound B-cell depletion [59] (Figure 1). Obinutuzumab has demonstrated some promise in B-cell depletion for desensitization in highly sensitized patients awaiting kidney transplant in the phase 1 theory trial; however, it has been associated with an inconsistent reduction in anti-HLA antibodies [60].

In the treatment of AMR, obinutuzumab has largely been reserved as an alternative anti-CD20 therapy for refractory cases or in the setting of rituximab intolerance and, to date, the two have not been compared directly. While the literature is limited, cases of AMR non-responsive to rituximab have reported a successful reduction in DSA titers and the stabilization of graft function [61].

Possible adverse events include infections, thrombocytopenia, infusion-related reactions, and cardiac events [62] (see Table 2 for a summary of the reviewed drugs).

### 5.3. Inebilizumab

Inebilizumab is a CD19 targeting agent. CD19 is expressed in B-cells; inebilizumab targets these B-cells and depletes them. It has been approved in 2020 for use in neuromyelitis [63]. It has been studied for use in highly sensitized patients awaiting first or second kidney transplants from deceased donors, but the study has been withdrawn due to no recruitment [64].

## 6. Conclusions

Conventional therapies for AMR are still not optimal, with high rates of graft loss leading to poor patient outcomes. Clearly, additional studies to define the optimal treatment of AMR are needed.

Newer therapies that target novel pathways in the AMR pathologic process are promising, but randomized studies are vital given the lack of randomized studies with adequate statistical power to compare the safety and efficacy of these novel therapeutics.

## Figures and Tables

**Table 1:** RCTs for prevention and/or treatment of AMR classified by mechanism of intervention.

Trial (NCT Number)	Intervention	Control Arm	Intervention Arm	Design/Status	Primary Endpoint	Follow-Up Duration
**Proteasome inhibition**						
**Bortezomib in AMR** **(NCT03737136)**	TPE-IVIG-RTX vs. TPE-IVIG-RTX and Bortezomib	10	10	Randomized double-blind Recruiting	Graft survival at 6 months	6 months
**IL-6 receptor blockade**						
**IMAGINE** **(NCT03744910)**	Clazakizumab 12.5 mg SC once every 4 weeks vs. matching placebo	175	175	Randomized, double-blind, multicentered Phase III Recruiting	Time to all-cause composite graft loss	5.5 years
**INTERCEPT** **(NCT04561986)**	Conventional therapy + tocilizumab vs. conventional therapies	25	25	Randomized, controlled Open-label Phase III Recruiting	Change from baseline in eGFR at 24 months	36 months
**CD38 blockade**						
**DARDAR** **(NCT04204980)**	Daratumumab dose escalation vs. full dose	13	13	Open-label, sequential assignment Phase I/II Recruiting	SAEs and AEs during the dose-escalation step for 21 months Intra-patients variation in cPRA at 0 and 6 months	21 months

**Table 2:** A summary of all medications reviewed in this article.

Name of Drug	Mechanism of Action	Use in Kidney Transplant	Type of Study	Participants	Efficacy Measures	Reported Side Effects
**Carflizomib**	Protesome inhibitor	Acute AMR	Clinical Trial	6 non-human subjects	DSAs, kidney rejection scores	Acute kidney injury, thrombocytopenia, infections
**Tocilizumab**	IL-6 receptor inhibitor	Desensitization	Phase I/II clinical trial	10	DSA titers, prevention of AMR	Infections, gastrointestinal perforation, elevation of transaminases
		Acute AMR	Clinical Trial	7	DSA titers	
		Chronic AMR	Multitude of Studies	36/15/10	DSA titers, histology, proteinuria	
**Clazakizumab**	IL-6 inhibitor	Refractory AMR, currently being studied for use in chronic AMR	Phase II single center open label study	10	eGFR, DSA titers, graft inflammation	Diverticulitis, pleural effusion, acute kidney injury
**Daratumumab**	Monoclonal antibody targeting CD38	Desensitization	Clinical trial	8 non-human subjects and 2 human subjects	DSA titers	Infusion-related reaction, volume overload, hypogammaglobulinemia, myelosuppression, gastrointestinal upset, infection
		Acute AMR	Multiple case reports	multiple	Graft function	
		Chronic AMR	Case report	2	DSA titers, graft function	
**Belimumab**	Anti-B lymphocyte simulator (BLyS)	Prevention of AMR	Phase II clinical trial	28	Comparison to standard of care results and infection rates	Gastrointestinal upset, dizziness, infection, depression, diabetes
**Imlifidase**	Recombinant cysteine protease	Desensitization	Multicenter clinical trial	39	Graft survival, patients survival, rates of AMR	Well tolerated, safety is currently being studied
**Obintuzumab**	Anti-CD20	Desensitization	Phase I clinical trial	24	Adverse events, B-Cell depletion	Infections, thrombocytopenia, infusion-related reactions, cardiac events
